# Observations on Chronic and Acute Rheumatism

**Published:** 1808-09

**Authors:** B. Carter


### *Observations on Chronic and Acute Rheumatism; By* Mr. B. Carter.

THIS disease, by the long and severe pains accompanying it, has much afflicted mankind, and frequently brought disgrace upon physicians. Its obstinacy, the difficulty of investigating its cause, and pointing out the indications of cure, render it a disease formidable in the annals of medicine.

Various have been the opinions of physicians on this disorder. We shall not undertake to give a history of the controversies on this subject, but shall briefly deliver what appears to be the most rational, towards defining and curing the disorder.

The rheumatism is by most authors divided into two species, the acute and chronical. The acute usually attacks the young and robust, and is always attended more or less with a phlogistic diathesis of the system. The chronical attacks the old, who have been formerly subject to the acute species. Dr. Cullen observes, that the limits between the acute and chronical rheumatism are not always exactly marked.

Though

Though the acute rheumatism nearly resembles the gout, yet in some respects it differs from it. It does not usually come on so suddenly as a fit of the gout, but for the most part gives the patient warning by a slow and gradual increase of pain. Neither is it fixed to one place, like the gout, but is distinguished by its frequent wanderings from place to place, accompanied with a sense of numbness. It seldom attacks the small joints, but is confined chiefly to the larger, the knees, hips, and shoulders. The acute rheumatism is generally attended with a continual fever, whereas the gout has periodical remissions. Like most of the pyrexia, it is preceded by a chilly fit, and a sense of cold. A febrile pulse, quick and hard, supervenes. The veins near the part affected swell, and a throbbing is felt in the arteries. By degrees the pain increases, and the patient suffers cruel tortures, which are increased upon the least motion. The sense of pain resembles that of a slow dilaceration of the parts, and commonly goes off by a swelling of the joints.

This disease is not wholly confined to the joints, it sometimes affects the limbs and other parts. It makes its attacks commonly in the spring and autumn, but is not confined to any season of the year. An exacerbation of all the symptoms takes place at night, when the patient is warm in bed. It most frequently attacks those whose employments subject them to alternations of heat and cold. A popular physician remarks, that the most difficult case of the rheumatism he ever met with, was that of a man whose employment was one half of the day in water, and the other half by the fire. It may be observed, that an attack of the rheumatism renders a person more liable to another, especially as Sydenham observes, if the first case has not been well treated. It attacks males more frequently than females.

The chronical rheumatism is much more frequently met with in practice than the acute; it is a disorder almost peculiar to the old and infirm, and is felt by them at irregular periods. This disease is rarely attended with mortality, but from its grievous symptoms, its frequent tendency to return and its troublesome pains, an effectual and permanent cure is much to be desired. But first let us endeavour to investigate its cause, before we attempt to point out the curative indication.

Concerning the cause of this disease, there have been many opinions, but we shall not attempt to give a history of the disputes of physicians on this subject. The late improvements in medicine teach us not to put implicit confidence in any name, how great soever. By this means is the knowledge of medicine to be advanced. What Dr. Cullen has written concerning the cause, appears to be unsatisfactory, though great praise is due to that gentleman for his accurate diagnostics. The causes of the rheumatism are undoubtedly debilitating. These are cold, and others, which when applied to the body, increase the excitability and diminish the excitement. The blood is not duly propelled to the extremities, it stagnates and degenerates into an acrid state, which exasperates the nerves and tendons, and produces pain, inflammation, and swelling.

Alternations of heat and cold appear evidently to be the proximate cause of the rheumatism. The rationale is thus explained. When the body is acted upon by any great stimulus, the sudden abstraction of it is attended with dangerous consequences. Thus the excitability being acted upon by the stimulus of heat, in a considerable degree, produces an accelerated motion in the fluids, and rouses up action in vessels which were before torpid. Now cold, which is an abstraction of stimulus, being suddenly applied, must arrest and detain a quantity of fluids in the vessels. By the application of cold, the action of the heart and arteries is weakened, the humours which are arrested in the extreme small vessels have a tendency to putrefaction, the juices become acrid and irritate the parts, whence proceed the inflammation, pain, and other symptoms of the rheumatism.

The reason why this disorder so frequently manifests itself in the bones and hard parts is, that the fluids find a greater resistance in the hard than in the soft parts, on account of the lesser diameters of their vessels, and hence are more liable to be obstructed. The reason why it affects principally the joints is, that in them, an equal number of vessels is contained in a smaller compass; hence the vessels themselves must be of smaller diameters in the joints than in the muscular parts, consequently there is a greater resistance made to the fluids propelled into them, and the impulse of the fluids causes a gradual dilaceration in their hard parts, and a consequent pain. To this may be added, that the joints are less covered with the cellular integument than any other part, whence they suffer most from the cold, as is evident in the knees. By this their excitement is diminished, and consequently a greater excitability takes place place, which renders them more liable to be affected by external causes.

Some physicians deny that an acrid matter can be the cause of rheumatism, and rank this and all others, among disorders of the solid parts. But with respect to the seat of diseases, whether in the fluids or solids, it is not worth while to contend, since the fluids and solids act reciprocally on each other. Some say it is impossible that any other than healthy humours should flow in the vessels. But in an ulcer, gangrene, or sphacelation, we see a manifest tendency of the humours to corruption. Upon the whole, we shall not be far from the truth, if we consider the rheumatism as a disorder of obstructed perspiration.

Having thus in a few words defined a rheumatism, I proceed to show the method of cure. The more accurate our knowledge is of the nature and causes of disease, the more rational will be the practice. It is in vain to expect a specific for this, or any other disorder. Whoever supposes that there is a medicine, an archæus, which, when taken into the stomach, or applied externally, will always certainly and immediately effect a cure; whoever is so unreasonable as to demand this, must expect disappointment. Human abilities are not however so limited, but that physicians can approximate towards a cure. In many cases, the pains never return again, and the disorder is radically cured; and frequently when the enemy cannot be vanquished, his attacks are parried in some degree, and mitigated or avoided by prudence.

In the acute rheumatism, if the patient be young and plethoric, venesection must be performed and repeated while the blood continues sizy. Administer a lenient cathartic every third day, and let the drink be barley water, acidulated with cremor tartar. Nitre is also recommended and the saline draughts. In short, the general treatment of this disorder must be similar to that of an inflammatory fever. Local applications may be varied, but I have found a blister the most efficacious. It should be kept open for some time. Emollient fomentations will sometimes remove a slight paroxysm. The parts may likewise be rubbed with volatile liniment, and leeches and cupping glasses occasionally applied. A free perspiration must be kept up, as on this depends, in a great measure, the cure. With this intention Dr. James's fever powders, or the essence of antimony, may be taken, with the decoct. guaiac. vel sarsaparill. and at night a bolus of camphor and opium.

In the chronical rheumatism venesection may be omitted,

-ted, unless the urgency of the case require it. An emetic or a cathartic is sometimes useful to unload the primæ viæ. Gum guiacum has been extolled as an excellent medicine in this disease. The patient should persevere in its use for some time. The essence of antimony may also be given, and the patient should wear flannel next his skin.

Venereal pains are sometimes mistaken for rheumatic. For these, mercury with the nitric acid will be found the most efficacious.

The dysentery and the rheumatism make frequent transitions from one to the other, which induced Dr. Akenside to think, that the matter and cause of both these disorders are the same.

A more generous diet than usual has frequently cured the rheumatism in poor people, after medicines had failed. Upon the whole, whatever tends to mend the habit, to improve the constitution, and to promote perspiration, as a generous diet, cheerfulness, exercise, the use of the flesh-brush, &c. will be found beneficial in the chronical rheumatism.

*No.* 68, *Hatton Garden,*
*August* 8, 1808.

